# Sexually transmitted pathogens in asymptomatic women at Rethabile clinic, Limpopo, South Africa

**DOI:** 10.4102/sajid.v39i1.618

**Published:** 2024-11-27

**Authors:** Dembe Mukavhanyedzi, Ivy Rukasha

**Affiliations:** 1 Department of Pathology, Faculty of Health Sciences, School of Medicine, University of Limpopo, Polokwane, South Africa; 2 Department of Microbiology, National Health Laboratory Service, Polokwane, South Africa

**Keywords:** sexually transmitted infections, asymptomatic, antenatal care, multiplex PCR, pregnant women

## Abstract

**Background:**

Health care for sexually transmitted infections (STIs) is often inadequate, especially for women, because of the asymptomatic nature of many STIs, which can lead to a false sense of health. Thus, there is limited data on the prevalence of STIs in pregnant women in low and middle-income countries.

**Objectives:**

The study aimed to determine the prevalence of STIs in asymptomatic pregnant women attending antenatal Rethabile Community Health Centre, Limpopo, South Africa.

**Method:**

A cross-sectional analysis of asymptomatic pregnant women at Rethabile Community Health Centre between March 2023 and November 2023 was conducted to determine the prevalence of seven STIs, detected from self-collected vaginal swab specimens using HAIN fluoroType STI-multiplex Polymerase Chain Reaction (PCR) test for nine targets covering seven major STIs.

**Results:**

The study found that *Ureaplasma urealyticum* was the most prevalent pathogen (43%) followed by *Chlamydia trachomatis* (41%), and *Trichomonas vaginalis* (10%). The less common pathogens detected were *Mycoplasma Genitalium* (5%) and *Neisseria gonorrhoeae* (2%).

**Conclusion:**

High STI prevalence among asymptomatic pregnant women at Rethabile Community Health Centre necessitates diagnostic screening over syndromic management because of a lack of reporting for symptoms.

**Contribution:**

The paper examines the epidemiology of STIs in Limpopo, South Africa, focusing on healthy, asymptomatic populations. It emphasises the need for laboratory screening, particularly in pregnant women, over empiric treatment because of high chances of missing infections.

## Introduction

Sub-Saharan Africa has the highest prevalence of sexually transmitted infections (STIs) compared to other regions in the world. The World Health Organization (WHO) has estimated that 40% of the global STI prevalence is in sub-Saharan Africa. South Africa, in addition to being the epicentre of the HIV epidemic in the world, also has the highest number of STIs in the region.^1,2^

Having STIs during pregnancy period has been linked with numerous adverse pregnancy and birth aftermaths including intrauterine death, preterm delivery and intrauterine growth retardation.^3^ Long-term morbidities such as cervical cancers, have also been linked with STIs.^4^ Sexually transmitted infections have also been associated with long-term morbidities which include chronic hepatitis, pelvic morbidities and cervical malignancies. Pregnant women who have gonococcal infections are at risk of passing the infection on to their unborn babies, which can result in ophthalmia neonatorum, a prominent cause of blindness.^5^

Conventionally, STI treatment is based on syndromic management to combat the influx of bacterial and viral STIs.^5^ Syndromic management is based on self-reported symptoms, physical signs and the clinician’s subjective assessment, which can occasionally be vague, unreliable or even deceptive, thus leading to failure in the detection of STIs in symptomatic participants, over-diagnosis and over-treatment of symptoms.^6^ The requirement for regular STI aetiological and antimicrobial resistance surveys is an important part of the syndromic management approach. This is to ensure that the flow charts and treatment algorithms can still treat most of the pathogens that cause STI syndromes.^7^ In addition, surveillance is crucial to detect the existence of STIs in the asymptomatic phase. The asymptomatic phase has an evolutionary advantage for the STI pathogens as it allows the existence of infection for longer.^8^ Thus, as a result, the true burden of STIs is unknown and underestimated. Therefore, laboratory diagnosis is crucial in preventing the spread of STIs. Apart from assisting in diagnosing symptomatic STI cases, laboratory diagnosis helps detect unusual cases, silent infections, and numerous infections.

In developing countries including South Africa, because of syndromic management of STIs, laboratory diagnosis of STIs has now become scarce because of low sample volumes resulting in loss of skill in most laboratories. As a result, the true burden of STIs in South Africa is limited. A study conducted in KwaZulu-Natal showed that STI prevalence of Herpes simplex virus type-2 (HSV-2) was 57.8%, syphilis was 1.6%, *Neisseria gonorrhoeae* was 2.8%, *Chlamydia trachomatis* was 7.1%, *Trichomonas vaginalis* was 9.0%, *Mycoplasma genitalium* was 5.5% and HIV was 36.3%.^9^ Studies conducted in Gauteng and Cape Town have shown STI prevalence of *C. trachomatis* to be 20%, followed by *T. vaginalis* (15%) and then *N. gonorrhoeae* (5.8%).^10,11^ However, there is partial information on the prevalence of STIs in Limpopo, leading to blind treatment following guidelines created using data from other provinces. Thus, the purpose of this study was to determine the prevalence of STI pathogens among pregnant women in Limpopo by simultaneously detecting a range of STI pathogens, namely, *C. trachomatis, N. gonorrhoeae, T. vaginalis, Mycoplasma hominis, M. genitalium, Ureaplasma urealyticum and Ureaplasma parvum,* using a multiplex syndromic panel PCR assay.

## Research methods and design

### Study design

A cross-sectional and prospective study was conducted among 202 different enrolled pregnant women at Rethabile Community Health Care Centre, Limpopo, South Africa.

### Study setting

The study was conducted on pregnant women attending a public sector antenatal clinic in an upscale suburban area in the Capricorn district. The clinic serves around 20 000 people of middle to higher socio-economic status.

### Study population and sampling strategy

The study was conducted at the Rethabile Community Health Centre, Polokwane, South Africa. Participants were selected using a convenience sampling method that focussed only on pregnant women who were available at antenatal clinic and were willing to participate in the study.

### Data collection and testing

Data collection was done using a questionnaire and specimen analysis. Participants were given a structured questionnaire written in English and Sepedi, the languages most people in the study area speak. Data on socio-demographic traits (age, marital status and educational attainment) and medical history (previous STI, HIV status and diabetic status) were then gathered.

### Specimen collection

Standard operating protocols were followed for the collection handling and processing of the materials. The participants self-collected samples using vaginal swabs, with the aid of instructions received from a trained midwife nurse and written on printed leaflets. The collected samples were then transported to the National Health Laboratory Service (NHLS), Medical Microbiology, Polokwane laboratory, within a few hours of collection. A multiplex syndromic panel PCR assay (HAIN Fluorotype STI Multiplex PCR) for qualitative detection of five sexually transmitted pathogens and two commensals was then used according to the manufacturer’s instructions. The assay contains an internal control to ensure extraction is efficient. A known positive control with all the pathogens is included in the kit to ensure PCR reactions took place and detection is accurate, and a mandatory negative control to control for contamination was found during the processing of the specimens.

### Data analysis

Data were recorded on Microsoft Excel and later imported to Statistical Package for the Social Sciences (SPSS) version 22.7 (SPSS Inc, Chicago, Illinois, United States) software for statistical analysis. Analysis was first carried out using univariate analysis for all the variables before being included in the final model. In this study, a patient was defined as being positive if a participant had at least one pathogen (*C. trachomatis, U. urealyticum, T. vaginalis, N. gonorrhoeae, M. genitalium*), while participants were defined as having a commensal if the participants had either *M. hominis* or *U. parvum*. Participants were recorded as being negative when they had neither a pathogen nor commensal. All variables significantly associated with being symptomatic at *p* < 0.25 in univariate logistic regression were included later for multivariate analysis. Chi-square statistical test was used to find the association between the socio-demographic characteristics of pregnant women and the prevalence of STIs. The level of significance was set at *p* < 0.05.

### Ethical considerations

Ethics approval to conduct the study was issued by the Turfloop Research Ethics Committee of the University of Limpopo (TREC/531/2023UG). Written informed consent was obtained from all individual participants involved in the study.

## Results

### Number of participants included in the study

**FIGURE 1:**
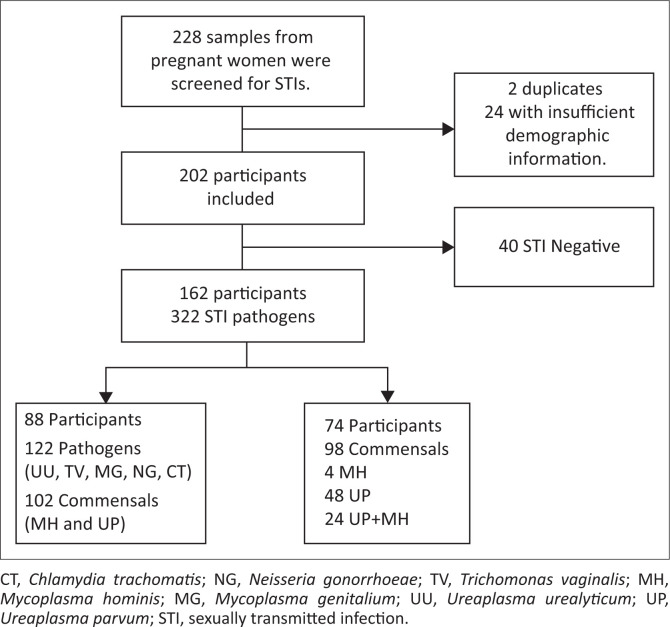
Flow diagram illustrating the samples included in the study.

A total of 228 consenting pregnant women from the age of 18 were included in the study. Unique identifiers which include surnames and names of participants along with their date of birth were used to remove duplicate samples. Only two samples were found to be duplicates, while 24 had insufficient information. Data from 202 participants met the inclusion criteria and thus were included for further analysis (Figure 1).

The median age of the 202 pregnant women was found to be 29 years (inter-quartile range [IQR] 26–33 years). The age group of 26–35 years had the highest number of participants with 128 (63%), followed by the age group of 18–25 years with 48 participants (24%) and the lowest number of participants (26; 13%) was in the 36–45-year age group. A higher proportion of the participants (134; 66%) had tertiary levels, while 64 (32%) and 2 (1%) had secondary and primary levels of education, respectively. Most of the participants (162; 80.2%) were single and 40 (19.8%) were married. A little more than half, that is, 114 (56.4%) of the participants were in the third-trimester. Of the 202 participants tested, 88 (43.5%) were positive for at least one STI pathogen and 74 (36.6%) had only commensals (98 commensals detected). In all, 122 different pathogens were detected from the 88 participants who had at least one pathogen, with 102 commensals also detected in these 88 participants.

**FIGURE 2:**
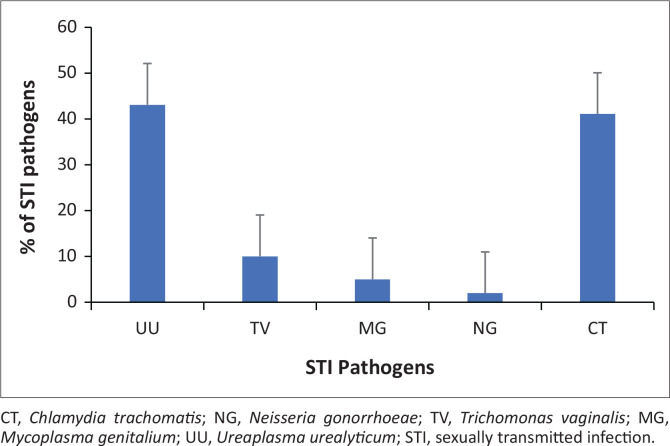
The distribution of sexually transmitted infection pathogens in pregnant women.

Figure 2 shows the distribution of pathogens among those that had a positive pathogen. Out of the 122 pathogens, the most common pathogenic agent detected was *U. urealyticum* (52; 43%) followed by *C. trachomatis* (50; 41%), and *T. vaginalis* (12; 10%). The less common pathogens detected were *M. genitalium* (6; 5%) and *N. gonorrhoea* (2; 2%).

The socio-demographic characteristics and distribution of STI pathogens among pregnant women are shown in Table 1. The percentage of women who tested positive for STIs increased with the gestation period; 0 (0%) of them did so in the first trimester and 44 (38.6%) in the third trimester. Gestation period and STI pathogen detection did significantly correlate with one another (*p* ≤ 0.001). Concerning age, the opposite was true. As age groups increased, the percentage of pregnant women who tested positive for STI pathogens decreased: 53 (41.4%) in the 26–35 years age group and 8 (30.8%) in the 36–45 years age group. However, there was no statistical significance in the relationship between age group and STIs (*p* = 0.125). Similarly, there was no statistically significant correlation found between any other socio-demographic factor and the presence of STI pathogens.

**TABLE 1:** Socio-demographic characteristics of pregnant women who attend antenatal clinic at Rethabile Community Health Centre in Polokwane, Limpopo province, 2023.

Variable	Category	Sexually transmitted pathogen				Total	%	*p*
		Yes		No				
		*n*	%	*n*	%			
Maternal age (years)		-	-	-	-	-	-	0.125
	18–25	26	54.2	22	45.8	48	23.8	-
	26–35	53	41.4	75	58.6	128	63.4	-
	36–45	8	30.8	18	69.2	26	12.9	-
Marital status		-	-	-	-	-	-	0.723
	Single	71	43.8	91	56.2	162	80.2	-
	Married	16	40.0	24	60.0	40	19.8	-
Education status		-	-	-	-	-	-	0.266
	Primary	2	50.0	2	50.0	4	2.0	-
	Secondary	28	43.8	36	56.3	64	31.7	-
	Tertiary	57	42.5	77	57.5	134	66.3	-
Gestation period		-	-	-	-	-	-	< 0.001
	First trimester	0	0.0	12	100.0	12	5.9	-
	Second trimester	43	56.6	33	43.4	76	37.6	-
	Third trimester	44	38.6	70	61.4	114	56.4	-

## Discussion

This study identified a high STI pathogen prevalence in self-reported asymptomatic pregnant women attending the antenatal clinic at Rethabile Community Centre. The current study represents rare studies that focussed only on self-reported asymptomatic pregnant women. In this study, the isolated true pathogenic agents included *M. genitalium, C. trachomatis, U. urealyticum, T. vaginalis and N. gonorrhoeae* similar to the results found in previous studies of pregnant women in sub-Saharan countries.^12^ Up to 43.5% of pregnant women tested positive for a common treatable STI pathogen in the selected participant population of 202. The gestational age of the patient was found to be the only significant associated risk factor for having an STI pathogen. Importantly, almost all participants reported not being infected with an STI and believed they were free of STIs. The participants were all convinced they were people who took care of their health and had sufficient STI knowledge. None of the participants were on treatment nor reported a need for STI screening.

The worrying high prevalence of STIs among asymptomatic women may be linked to poor knowledge of STIs and the general asymptomatic nature of these pathogens as previously reported. The asymptomatic phase of STIs is the favoured stage of the existence of STIs due in part to the evolutionary advantage because existence with severe symptoms will lead to people seeking medical help or death, which will limit the ability to be passed to another humans. Thus, there is likely selective pressure for STIs to produce few or no symptoms for a long period or the entire duration of the infection.^8^

The prevalence of *T. vaginalis* in this study was found to be 10%. This was similar to the prevalence found in studies conducted in Nigeria (Ilorin) in which *T. vaginalis* was 9.7%.^13^ The Nigerian study had results similar to the current study; this may be because of populations consisting of mostly tertiary students attending college in an urban area. However, the studies conducted in Durban, Cape Town and Gauteng showed slightly higher prevalences of *T. vaginalis* ranging between 15% and 18%.^10,11,14^ These high prevalences may be influenced by the setting of these studies; they were conducted in rural areas and research suggests that individuals with limited education opportunities are more likely to have STIs because they might have difficulty in understanding health materials, including information about STIs, condoms and safe sex practices, and therefore they are mostly undiagnosed and untreated until screening measures are put in place.^15^

*Neisseria gonorrhoeae* was one of the STIs that had the lowest prevalence of 2%. This result is comparable to a previous study conducted in the Tshwane district and Cape Town with a prevalence of 4.2%.^11^ The prevalence of *N. gonorrhoeae* in a study that was conducted in Pretoria had a slightly higher prevalence of 6.9%. Pretoria is the densely populated city in South Africa, and has been markedly affected by both the HIV and STI epidemic.^3^ Another study that was conducted in Brikama, Gambia had a prevalence of 1.8%.^16^ This very low prevalence of gonorrhoea is attributed to the fact that data were collected from asymptomatic participants. Generally, gonorrhoea is a highly symptomatic infection. This means that persons affected tend to seek medical care within 48 h of the infection and hence almost all the participants tested negative for gonorrhoea.^17,18^

The prevalence of *C. trachomatis* in this study was found to be 41.0%, which was different from the prevalence in studies conducted in China and Tanzania with 10.8%, and 11.4%, respectively.^19,20^ These studies were conducted in a different setting (semi-urban area) to our study; hence, those studies have different prevalences of STIs. However, some studies showed a lower prevalence ranging from 0.8% to 8.6%.^21,22,23^ These studies were conducted in middle-income countries, Tshwane district, Cape Town and sub-Saharan Africa. These settings are more developed than Polokwane; hence, they produce lower prevalences of *C. trachomatis*. Other studies, conducted in Kenya (Kilifi), South Africa and KwaZulu-Natal, were found to display higher prevalences ranging from 26.1% to 46.3% than the ones we found in our studies.^14,24^ Generally, the prevalence and incidence of STIs vary across societies and sub-populations as defined by age, gender, race and ethnicity, and socioeconomic status.

Genital mycoplasmas (*U. urealyticum, M. genitalium, U. parvum, M. hominis*) are sometimes referred to as commensals and are mostly found in the respiratory, urinary and reproductive tracts. However, studies have shown that these bacteria are sexually transmitted and can be linked with STIs.^25^ It is worth noting that there is much controversy surrounding the pathogenicity of *Ureaplasma* spp. as an STI. The controversy has led differentiation of *Ureaplasma* into two species: *U. parvum* and *U. urealyticum*. According to several studies, *U. parvum* is a common organism that is not harmful and has a prevalence comparable to or lower than controls. However, *U. urealyticum* has been more significantly associated with symptomatic urogenital infections.^26,27^

The current study findings showed a higher prevalence (50%) of STIs in people with primary education than those with secondary level (44%) and tertiary level education (43%), although the difference was not statistically significant. However, most studies show a higher prevalence of STIs at the secondary level than at the tertiary level.^10,28^ High educational level has been shown to be positively associated with higher age of sexual intercourse and sexual health knowledge; and had a lower risk of unintended pregnancy and STIs.^3,29^

### Strength of the study

In this study, we used molecular-based techniques to detect the STIs, which have high specificity and sensitivity compared to conventional testing methods such as microscopy and culture assays. All samples were tested simultaneously to detect STIs using a multiplex panel on the same platforms eliminating the issue of variability brought about by different transport and storage conditions together with the laboratory assay environment resulting in increasing the compatibility which makes the results more reliable. The use of vaginal swabs instead of urine makes the results to be more reliable as urine may be less concentrated which might tamper with the detection of the STI causative agent and the results.

### Limitations of the study

This is a single-centre study; thus, the results of this study may not generalise across all geographic areas in Limpopo or South Africa. The study included self-collected vaginal swabs; even though printed leaflets on how to do self-collection of vaginal swabs were administered to the participants, some participants may have not collected the sample, thus leading to the wrong presentation of negative results. However, studies have shown statistically comparable results between self-collected swabs and physician-collected swabs.^28,29^ The study includes a low sample size because of the overall number of people who were attending antenatal clinics in the study area during the study time. The kit that was used in this study only tested for five STIs and two commensals, namely, *U. parvum and M. hominis* and did not test syphilis; thus, results for syphilis were not available.

## Conclusion

In conclusion, we found a high STI pathogen prevalence among pregnant women who self-reported to be free of STIs. Thus, the study supports the screening of multiple STIs in both symptomatic and asymptomatic pregnant women as part of prenatal care in South Africa.
